# Correlation analysis between hand hygiene product consumption trends and healthcare-associated infections in a tertiary comprehensive hospital in Yunnan Province, China (2015–2024)

**DOI:** 10.1016/j.infpip.2026.100594

**Published:** 2026-09-25

**Authors:** Hongping Pan, Chao Liang, Hongjuan Zhu, Hailan Fan, Yu Huang

**Affiliations:** a Department of Hospital Infection Management, The People’s Hospital of Yuxi City, The Sixth Affiliated Hospital of Kunming Medical University, Yuxi, Yunnan, China; b Dermatology & STD Department, The People’s Hospital of Yuxi City, The Sixth Affiliated Hospital of Kunming Medical University, Yuxi, Yunnan, China; c Nursing Department, The People’s Hospital of Yuxi City, The Sixth Affiliated Hospital of Kunming Medical University, Yuxi, Yunnan, China

**Keywords:** Hand hygiene product, Consumption, Healthcare-associated infection, Correlation

## Abstract

**Background:**

Hand hygiene (HH) is an effective, safe, and cost-effective measure for preventing healthcare-associated infections (HAIs); improving HH compliance can reduce HAI rates. HH product consumption is one of the indicators used to assess HH performance, and analysing this consumption can support the enhancement of HH management.

**Aim:**

To analyse HH product consumption trends and their correlation with HAIs in a Chinese tertiary hospital from 2015 to 2024.

**Methods:**

Data on HH product consumption, patient-days, and HAI cases were retrospectively collected. Consumption per patient-day (mL/PD) was calculated.

**Findings:**

From 2015 to 2024, overall median HH consumption was 13.07 mL/PD, varying significantly across departments (*P < 0.001*). Intensive care unit/neonatal intensive care unit (ICU/NICU) used more alcohol-based hand rub (ABHR) than soap after 2020, whereas other wards used more soap. HH product consumption peaked in 2021 (15.99 mL/PD). Consumption changes were notably significant in the ICU and NICU. Annual HH product consumption varied significantly (*P < 0.001*) and increased significantly post-coronavirus disease 2019 (COVID-19) (*P < 0.001*), mainly driven by hand rub. Hospital-wide HAI incidence showed a negative correlation with HH product consumption (*r =* −0.640*, P =* 0.046). An increase of 1 mL/PD in HH product consumption was associated with a decrease of 0.138% in the HAI case incidence rate.

**Conclusion:**

HH product consumption varied by department and initially increased and then declined. Greater HH product use was associated with lower HAI rates. Targeted interventions are needed to sustain HH practice improvements.

Healthcare-associated infections (HAIs) contribute significantly to patient morbidity, mortality, and economic burden on healthcare systems and are recognized globally as a major public health issue [1]. Contaminated hands of healthcare workers (HCWs) are a crucial vector for the transmission of HAIs. In 1847, Semmelweis demonstrated that hand washing with chlorinated lime solution effectively reduced maternal mortality [2]. Hand hygiene (HH) effectively curbs the transmission of *methicillin-resistant Staphylococcus aureus*, *Clostridium difficile*, and *vancomycin-resistant Enterococcus*, making it a critical measure for preventing HAIs and ensuring patient safety [3]. Studies indicate that investment in HH offers favourable cost-effectiveness, with an estimated saving of approximately US$16.5 in healthcare expenditures for every US$1.0 invested [4]. However, HH compliance among healthcare institutions worldwide remains suboptimal, estimated at approximately 40% in high-income countries and below 20% in low-income countries [2]. Multi-modal HH strategies combined with effective intervention packages can effectively improve compliance [2]. Monitoring HH product consumption helps enhance HCW HH compliance [5], and it is also recognized as one of the key surrogate measures for assessing compliance [6]. This study monitored HH product consumption over a decade in a tertiary comprehensive hospital, analysing the composition of products used in different departments, consumption trends, and correlations with HAIs, aiming to provide evidence for formulating HH strategies and reducing HAI occurrence.

## Materials and methods

### Data sources

A tertiary comprehensive hospital located in Yuxi City, Central Yunnan Province, China, with 1500 beds, was selected. This hospital has monitored HH product consumption since 2015. Data were sourced from the hospital's logistics management system, financial system, and hospital infection surveillance system for the period 2015–2024.

### Methods

Infection control professionals retrieved quarterly data on the quantity of liquid soap and alcohol-based hand rub (ABHR) requisitioned by all hospital departments from the logistics and financial systems. Data on the total number of inpatients, total patient-days, and number of HAI cases per department per quarter were obtained from the hospital infection surveillance system.

Consumption of HH products per patient-day = HH product consumption/patient-days.

Consumption of HH products per patient = HH product consumption/number of patients.

HAI rates were analysed using the HAI case incidence rate:

HAI case incidence rate = (Number of new HAI cases among inpatients during a specified period/total number of inpatients during the same period) × 100%.

### Statistical analysis

Statistical analysis was performed using SPSS 20.0 (IBM Corp., Armonk, NY, USA). HH product consumption per patient-day and per patient were calculated. For comparisons of measurement data with skewed distributions between groups, the Kruskal–Wallis *H* test was used. For comparisons between two time periods (pre- and post-coronavirus disease 2019 [COVID-19]), the Wilcoxon rank-sum test was applied. The correlation between HH product consumption and HAI incidence rates was analysed using Pearson correlation. A *P*-value < 0.05 was considered statistically significant. Graphs were generated using RStudio (2025.9.2.0, Posit Software, PBC).

## Results

### General information

Over the 10-year study period (2015–2024), data were included from 1 comprehensive intensive care unit (ICU), 1 neonatal intensive care unit (NICU), 4 general wards + intensive care areas (ICA: respiratory medicine, cardiology, neurology, and neurosurgery), 6 general internal medicine wards (endocrinology, nephrology, gastroenterology, oncology, haematology, and geriatrics), and 13 general surgery wards (otorhinolaryngology, stomatology, urology, pain management, ophthalmology, orthopaedics, general surgery, breast surgery, dermatology, emergency surgery, gynaecology, obstetrics, and thoracic surgery). Nine departments were excluded due to a short establishment time or discontinuous data (emergency internal medicine, emergency intensive care unit, rehabilitation medicine, general medicine, cardiovascular surgery, paediatric internal medicine, radiotherapy, infectious diseases, and clinical psychology).

### HH product consumption

During 2015–2024, the overall hospital consumption of HH products was 13.07 mL/PD, comprising liquid soap consumption of 7.32 mL/PD and ABHR consumption of 5.75 mL/PD. Significant differences were found among departments in total consumption, liquid soap consumption, and ABHR consumption (*H = 1205.312; 620.758*; *584.748, all P < 0.001*). Consumption was highest in the ICU (101.92 mL/PD), followed by the NICU (24.44 mL/PD), general wards + ICA (13.62 mL/PD), general internal medicine (11.17 mL/PD), and general surgery (11.09 mL/PD). Regarding product composition, ABHR consumption surpassed liquid soap consumption in the ICU and NICU after 2020; in other departments, liquid soap consumption remained slightly higher than ABHR consumption (Table I, Figure 1).

**Table I:** Consumption of HH products in different departments

Ward	Total inpatients (*N*)	Patients- days (d)	Total consumption (mL)	Consumption per patient (mL)	Consumption per PD (mL/PD)	Liquid soap			ABHR		
						Consumption (mL)	Consumption per patient (mL)	Consumption per PD (mL/PD)	Consumption (mL)	Consumption per patient (mL)	Consumption per PD (mL/PD)
ICU	7900	50,656	5,162,964	653.54	101.92	2,659,500	336.65	52.50	2,503,464	316.89	49.42
Gen. ward + ICA	166,446	1,253,133	17,073,328	102.58	13.62	9,644,000	57.94	7.70	7,429,328	44.64	5.93
General internal med.	139,409	1,167,733	13,040,000	9.35	11.17	7,272,000	52.16	6.23	5,768,000	41.37	4.94
General surgery	359,329	2,495,972	27,685,320	76.72	11.09	16,239,600	45.19	6.51	11,445,720	31.85	4.59
NICU	22,131	173,946	4,250,500	192.06	24.44	1,844,500	83.34	10.60	2,406,000	108.72	13.83
**Total**	695,215	5,141,440	67,212,112	96.64	13.07	37,659,600	54.17	7.32	29,552,512	42.51	5.75

HH, hand hygiene; ICU, intensive care unit; NICU, neonatal intensive care unit; ABHR, alcohol-based hand rub.

**Figure 1:**
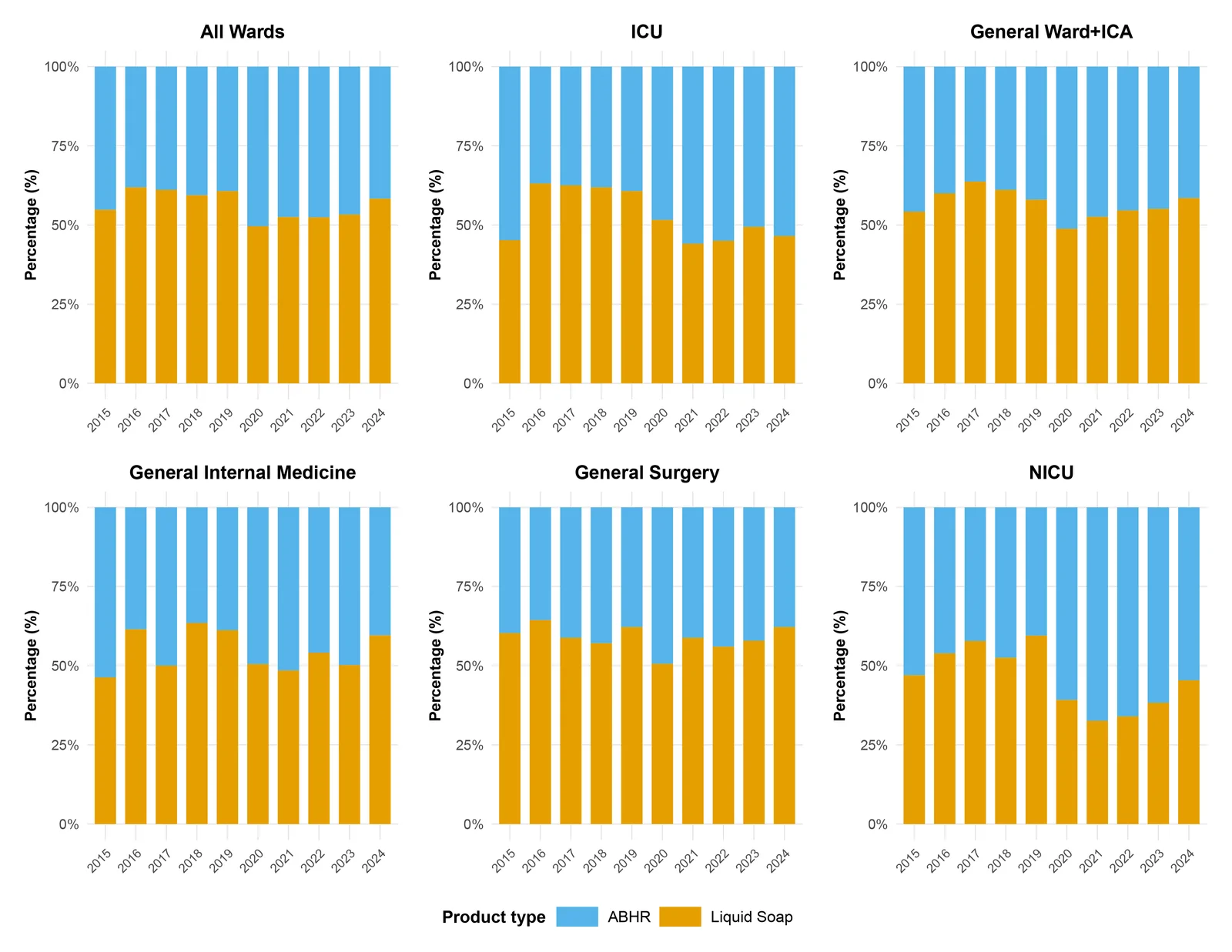
HH products consumption proportion by ward (2015–2024). HH, hand hygiene.

### Annual HH product consumption

Hospital-wide and departmental HH product consumption showed an increasing trend followed by a decline from 2015 to 2024. Two growth peaks were observed in 2016 and 2020. The hospital-wide consumption growth rate was 42.2% in 2016 and 22.9% in 2020. Consumption began to decline after 2022. The highest annual hospital-wide consumption occurred in 2021, reaching 15.99 mL/PD (liquid soap: 8.39 mL/PD; ABHR: 7.59 mL/PD). ICU consumption peaked in 2020 at 122.73 mL/PD (growth rate: 32.4%). NICU consumption peaked in 2022 at 43.11 mL/PD (growth rate: 44.1%), with liquid soap consumption showing a continuous increasing trend. Significant differences were observed across years for total HH product consumption, liquid soap consumption, and ABHR consumption (*H =* 313.959; 167.487; 145.165*, all P < 0.001*) (Table II, Figure 2, Appendix A. Figure S1).

**Table II:** Annual HH product consumption

Year	Total inpatients (*N*)	Bed days (days)	Total consumption (mL)	Consumption per patient (mL)	Millilitres per patient-day (mL/PD)	Liquid soap			ABHR		
						Consumption (mL)	Consumption per patient (mL)	Millilitres per patient-day (mL/PD)	Consumption (mL)	Consumption per patient (mL)	Millilitres per patient-day (mL/PD)
2015	54,050	488,092	4,480,500	80.73	8.94	2,457,000	45.46	5.03	2,023,500	37.44	4.15
2016	58,601	462,964	5,886,500	100.45	12.71	3,645,000	62.20	7.87	2,241,500	38.25	4.84
2017	61,415	483,038	5,958,500	97.02	12.34	3,642,000	59.30	7.54	2,316,500	37.72	4.80
2018	63,668	489,326	5,485,600	86.16	11.21	3,260,100	51.20	6.66	2,225,500	34.95	4.55
2019	64,595	493,282	6,164,000	95.43	12.50	3,741,000	57.91	7.58	2,423,000	37.51	4.91
2020	64,776	470,490	7,136,012	111.57	15.36	3,539,500	54.64	7.52	3,596,512	55.52	7.64
2021	72,871	513,343	8,206,000	112.61	15.99	4,307,500	59.11	8.39	3,898,500	53.50	7.59
2022	82,102	549,501	8,096,000	98.61	14.73	4,245,500	51.71	7.73	3,850,500	46.90	7.01
2023	85,212	587,154	7,705,000	90.42	13.12	4,106,500	48.19	6.99	3,598,500	42.23	6.13
2024	87,925	604,250	8,094,000	92.06	13.40	4,715,500	53.63	7.80	3,3785,00	38.42	5.59
Total	695,215	5,141,440	67,212,112	96.64	13.07	37,659,600	54.17	7.32	29,552,512	42.51	5.75

HH, hand hygiene; ABHR, alcohol-based hand rub.

**Figure 2:**
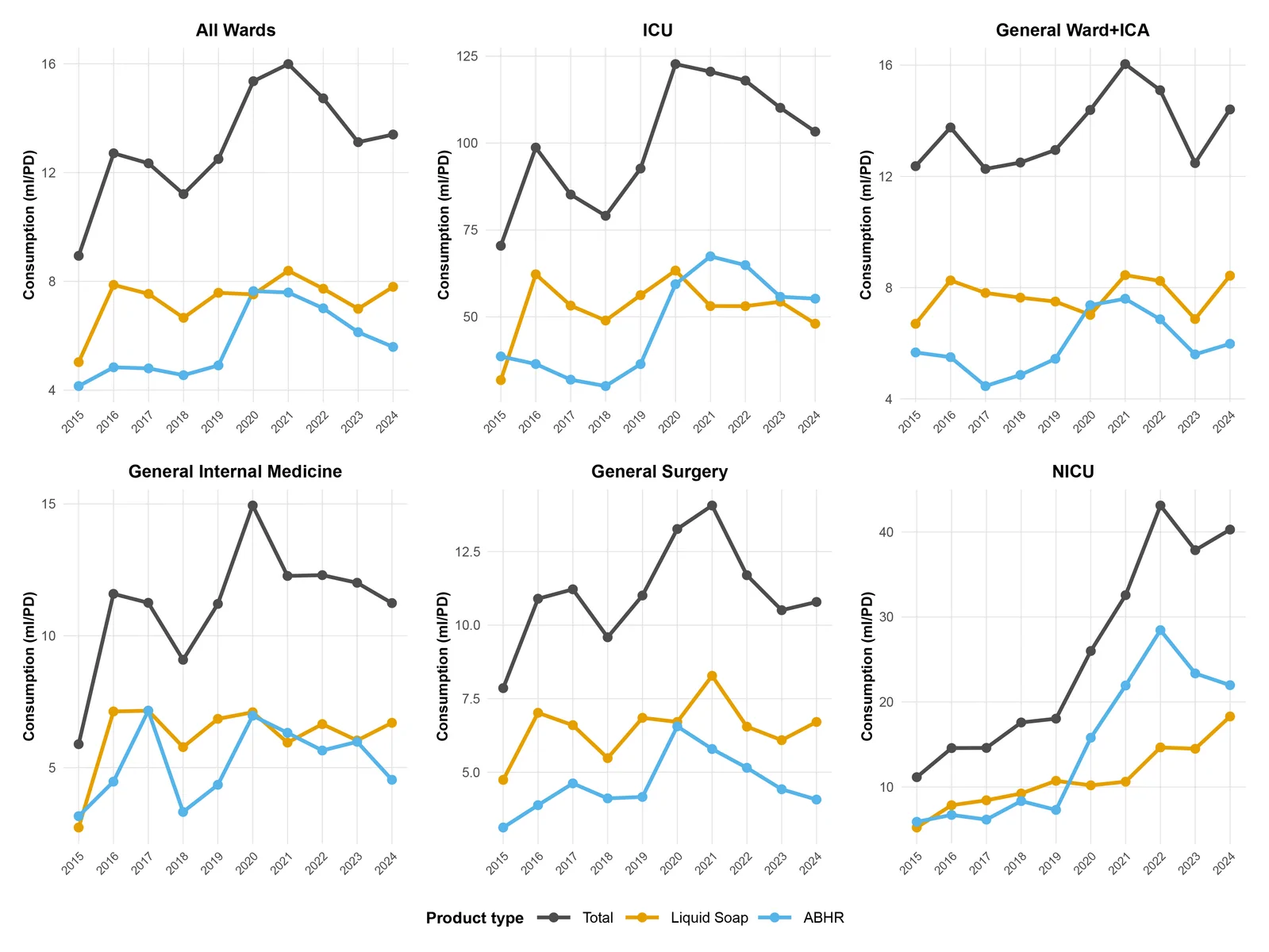
HH products consumption trends by ward (2015–2024). HH, hand hygiene.

### HH product consumption before and after the COVID-19 pandemic

Following the COVID-19 pandemic, hospital-wide consumption of total HH products, liquid soap, and ABHR increased by 24.21 mL/PD, 4.13 mL/PD, and 23.30 mL/PD, respectively. Statistically significant differences were found between the prepandemic and postpandemic periods for total HH product consumption and ABHR consumption (*Z =* −14.978; −10.325*; P < 0.001*), but not for liquid soap consumption (*Z =* −0.093*; P = 0.926*) (Table III).

**Table III:** HH product consumption before and after the COVID-19 pandemic (mL/PD)

HH products	2015–2019		2020–2024		Difference value	*Z*	*P*
	M	Q1-Q3	M	Q1-Q3			
Total consumption	79.09	13.76–92.69	103.30	32.57–120.55	24.21	−14.978	<0.001
Liquid Soap consumption	48.97	7.86–56.26	53.10	10.20–54.37	4.13	−0.093	0.926
ABHR Consumption	31.95	6.17–36.45	55.25	21.94–64.90	23.30	−10.325	<0.001

HH, hand hygiene; COVID-19, coronavirus disease 2019; ABHR, alcohol-based hand rub.

### Correlation between HH product consumption and HAIs

From 2015 to 2024, the hospital-wide HAI case incidence rate decreased from 2.20% to 0.76%. General internal medicine and general surgery wards showed an overall downward trend in HAI incidence rates. The HAI case incidence rate for the ICU、NICU, and general wards + ICAs, showed fluctuating trends (Appendix A: Table S1, Figure S2). A negative correlation was found between the hospital-wide HH product consumption per patient-day and the HAI case incidence rate (*r = −0.640, P = 0.046*). An increase in HH product consumption corresponded to a decline in the HAI case incidence rate. (Figure 3). Linear regression analysis indicated that HH product consumption had a negative impact on the HAI case incidence rate (*β =* −0.640*, t =* −2.356*, P =* 0.046). The unstandardized regression coefficient was −0.138 (*SE =* 0.058). The regression model was statistically significant (*F(1, 8) =* 5.553*, P =* 0.046), explaining 41.0% of the variance (*R*^*2*^ *= 0.410, adjusted R*^*2*^ *= 0.336*). The final regression equation was: $\hat{y}$ = 3.220 - 0.138χ, i.e. HAI case incidence rate = 3.220 - 0.138 × HH product consumption (mL/PD). An increase of 1 mL/PD in HH product consumption was associated with a decrease of 0.138% in the HAI case incidence rate.

**Figure 3:**
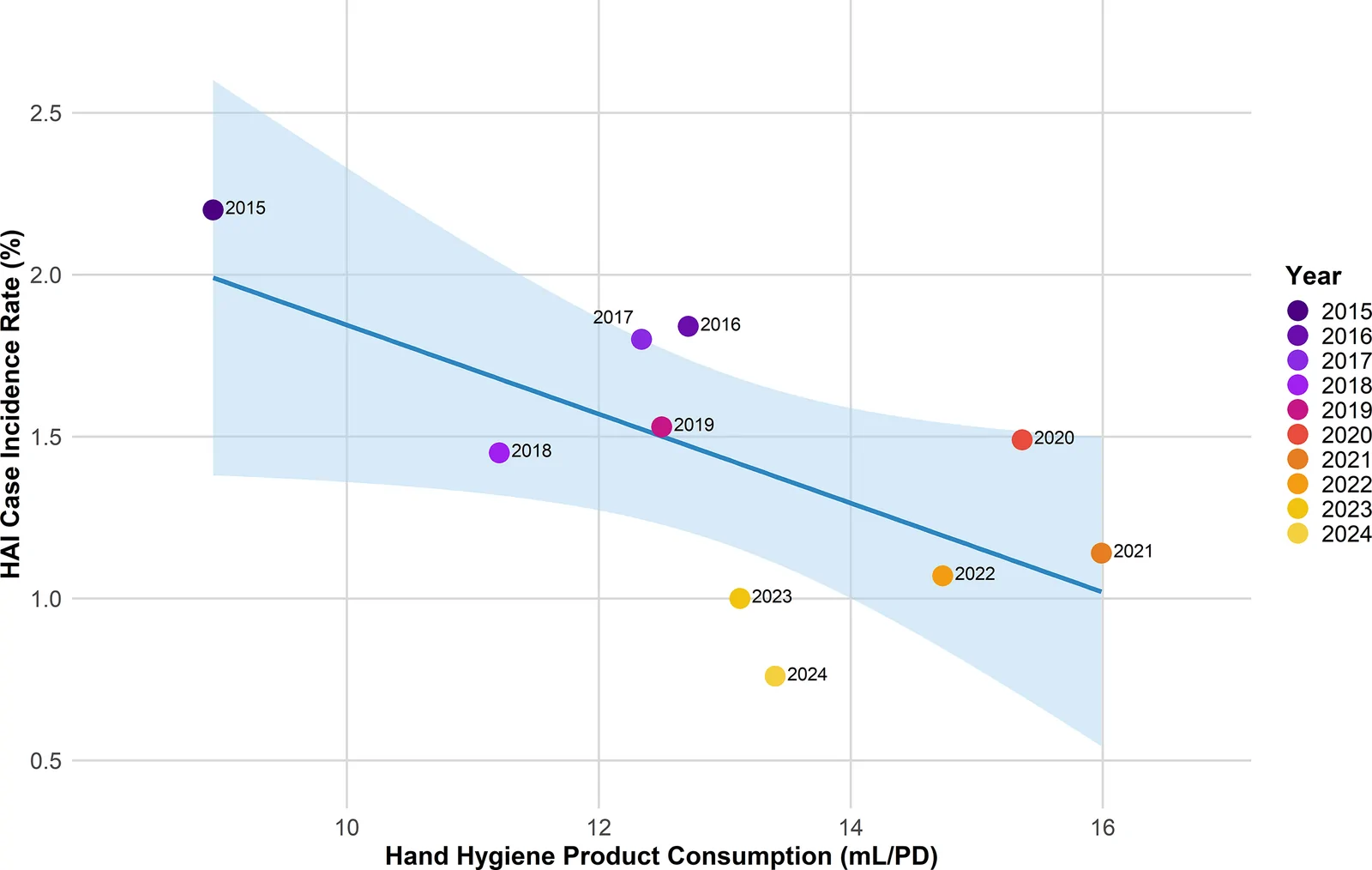
Relationship between HH product consumption and hospital-wide HAI case incidence rate (2015–2024). HH, hand hygiene; HAI, healthcare-associated infections.

The consumption of HH products in the NICU was positively correlated with the HAI case incidence rates (*r =* 0.762*, P =* 0.010). No significant correlation was found between consumption and HAI case incidence rates in the ICU (*r =* 0.624*, P =* 0.054), general wards + ICA (*r = −*0.467*, P =* 0.174), general internal medicine (*r = −*0.467*, P =* 0.174), and general surgery (*r =* 0.261*, P =* 0.467).

## Discussion

Hand hygiene product consumption is an intuitive and easily accessible surrogate marker and has been incorporated into monitoring systems in some regions and countries [6,7]. Since 2006, France has made it a mandatory quality indicator in annual public reports [6]; Germany includes it in routine healthcare facility surveillance data [8]; and the Shanghai Hospital Infection Control Center in China has included it in its regional monitoring programme since 2008 [9]. Studies have shown that HH product consumption is positively correlated with HH compliance [10] and can serve as a complementary indicator to traditional direct observation [11]. Feedback based on this metric can improve the visibility of consumption and motivate HCWs to perform HH [12].

In this study, the hospital-wide average consumption from 2015 to 2024 was 13.07 mL/PD (liquid soap: 7.32 mL/PD; ABHR: 5.75 mL/PD). This figure is below the World Health Organization (WHO) Hand Hygiene Self-Assessment Framework target of 20 mL/PD [11] and significantly lower than the average consumption of 55–60 mL/PD reported in a study covering 97 healthcare institutions across 28 countries in three WHO regions (Asia–Pacific, Europe and Latin America) from 2010-2021 [10], as well as the 41.63 mL/PD reported in Italy [13]. However, it is similar to reports from some Asian countries (Sri Lanka, China, and Thailand) where average ABHR consumption is <20 mL/PD [10].

Substantial differences in HH product consumption per patient-day existed among departments. Consumption was highest in the ICU (101.92 mL/PD), markedly higher than in other wards (NICU: 24.44 mL/PD, general wards + ICA: 13.62 mL/PD, general internal medicine: 11.17 mL/PD, general surgery: 11.09 mL/PD). ICU consumption falls within the recommended range for intensive care settings (100–160 mL/PD) [10], is higher than the average for European ICUs (66 mL/PD) [14] and Italian ICUs (73.93 mL/PD) [15], and is consistent with findings from domestic tertiary hospital studies showing the highest consumption in ICUs [16]. The specific ICU consumption (liquid soap: 52.50 mL/PD; ABHR: 49.42 mL/PD) exceeds the control requirements of some domestic ICUs (ABHR: >10 mL/PD, liquid soap: >20 mL/PD) [16]. The higher workload and greater number of HH opportunities inherent in intensive care likely drive this elevated consumption.

Barriers to HH product use include product characteristics, personal factors and organisational factors [12]. Interventions such as increasing the density of hand rub dispensers at patient bedsides in Switzerland [17] and providing better-tolerated HH products in Sierra Leone [18] have successfully increased consumption. Due to its convenience and speed, ABHR is increasingly used in healthcare settings [19]. Notably, in this study, except for the ICU and NICU, liquid soap consumption remained higher than ABHR consumption in all other departments. Research by Calcagni Nicolas *et al.* [12] in France found that some HCWs perceived soap as gentler and more effective, preferring it over ABHR, which they felt left a ‘false’ impression of clean skin. Therefore, further analysis is needed within institutions to identify factors influencing HH product use across different departments in order to develop targeted interventions.

Over the ten-year period, two consumption peaks were observed. The first peak occurred in 2016 (growth rate 42.2%), coinciding with the hospital's implementation of measures that included publicising departmental consumption data combined with weekly HH audits by infection control staff. This finding is similar to that of Fujita R *et al.* [5] in Japan, where monitoring consumption alongside direct observation significantly increased product use. Accumulating evidence supports multi-modal interventions-including staff education/training, system change, hospital reminders, direct observation with feedback, and promoting a patient safety climate-to enhance HH compliance [20].

The second consumption peak occurred in 2020, coinciding with the COVID-19 pandemic. After the pandemic, total consumption increased by 24.21 mL/PD, liquid soap by 4.13 mL/PD, and ABHR by 23.30 mL/PD, reflecting global trends [2] and demonstrating an unprecedented uniformity in HH awareness among healthcare providers during the COVID-19 crisis [21]. This likely stemmed from heightened self-protection awareness, psychological stress, and improved HH habits during the emergency [21,22]. However, this sharp increase was not sustained in the long-term; consumption declined after 2022, consistent with findings from Chebenová Vanesa *et al.* [7] showing a postpandemic decrease. The prominence of HH diminished over time, and without sustained monitoring to maintain institutional focus, compliance drifted back toward baseline levels [23]. Although pandemic-era training enhanced knowledge, without concurrent monitoring and feedback, the translation of knowledge into consistent practice (the ‘know-do gap’) persists. Achieving sustained improvement in HH compliance necessitates continuous professional education and comprehensive multi-modal strategies [7].

Global studies have examined the impact of HH interventions on HAIs. Research by Helena Ojanperä *et al.* [24] in Finland and Ding Jiayan *et al.* [16] in China demonstrated negative correlations between HH compliance rates and HAI rates (*r* = −0.48 and −0.369, respectively). Solange Angelina Bruchez *et al.* [25] in Brazil reported that following HH interventions in an adult ICU, ABHR use increased by 132% and the infection rate decreased by 22.6%. Didier Pittet *et al.* [19] in Switzerland showed that a HH promotion programme improved compliance from 48% to 66%, increased hand rub consumption from 3.5 mL/PD to 15.4 mL/PD, and concurrently reduced transmission rates of multi-drug-resistant organisms. Chia Yin Chong *et al.* [26] in Singapore reduced rotavirus infection rates by increasing HH product consumption from 82.6 mL/PD to 109.2 mL/PD through improved compliance. Direct observation of HCW HH compliance, a common intervention component, is susceptible to the Hawthorne effect and observer bias. Researchers have explored methods such as covert observation or electronic video surveillance with smart monitoring as complements [7,13,25]. The present study analysed and evaluated the direct, objective, and easily obtainable metric of HH product consumption in relation to HAI occurrence. The results show a negative correlation between hospital-wide HH product consumption per patient-day and the HAI case incidence rate; an increase of 1 mL/PD in HH product consumption is associated with a 0.138% decrease in the hospital-wide HAI case incidence rate. This suggests that increasing HH product usage during clinical care may help reduce HAIs, and that HH product consumption may serve as a valuable reference indicator for infection control evaluation in healthcare institutions.

It is noteworthy that the ward-stratified analysis of HH product consumption and HAIs showed a positive correlation in the NICU, while no correlation was found in the general wards + ICA, general internal medicine, or general surgery wards. This finding contradicts the conventional expectation that ‘increased HH consumption reduces HAIs’, suggesting that in real-world clinical practice, HAIs are influenced by confounding factors, and HH product consumption is only one of several associated factors. Therefore, such analyses should fully consider other confounders. For example, patient severity of illness is an important factor affecting HAIs: patients admitted to the ICU with higher severity scores have a greater infection risk [27]. Neonates, owing to their immature immune systems, are at high risk for HAIs and are prone to nosocomial outbreaks [28]. A study by Yang YiMei *et al.* [29] in China reported that approximately one-third of preterm infants with a gestational age of less than 32 weeks develop HAIs due to incomplete immune development, and the use of invasive devices such as Peripherally Inserted Central Catheter (PICC) further increases the infection risk. In addition, high ICU bed occupancy rates significantly increase HAI risk; prolonged ICU stay and longer duration of invasive device use are both independent risk factors for HAIs [30]. Adjustments in antimicrobial stewardship (AMS) strategies also influence infection rates. A systematic review by Shuayb Sikiru *et al.* [31] in Nigeria reported that implementing AMS programmes effectively reduces HAIs and antimicrobial resistance in both high-income and low- and middle-income countries; perioperative AMS implementation in African surgical settings reduced HAI risk by one-third. The staffing level of infection prevention and control personnel should not be overlooked; the number and experience of dedicated staff are inversely associated with HAI rates – more staff and greater experience correlate with lower HAI rates [32]. The use of personal protective equipment (PPE) also plays an important role. A study by Andreas Ambrosch *et al.* [33] in Germany showed that mandatory full-time masking of medical staff during influenza seasons reduced nosocomial influenza rates by 50%, effectively protecting patients from hospital-acquired influenza infection and death. The outbreak of the COVID-19 pandemic has also significantly altered the epidemiology of HAIs: Voinea Corina *et al.* [34] in Romania reported an increase in ‘Clostridioides difficile’ infection rates in the postpandemic period, linked to repeated antimicrobial use and patient immunosuppression; during the pandemic, elective surgeries were reduced, and aseptic techniques were reinforced, leading to lower infection rates, which subsequently rose after surgical services resumed. Environmental cleaning is another non-negligible factor. Pathogens can survive for long periods in healthcare environments, and environmental transmission is a major route for HAIs; improving and intensifying environmental cleaning can reduce HAIs [35,36].

In summary, the occurrence and fluctuation of HAIs are influenced by a complex interplay of multiple factors, including patient severity, invasive procedures, ICU occupancy, AMS strategies, staffing levels, PPE use, outbreak periods, and environmental cleaning. When analysing such data, caution must be exercised regarding the effects of confounding factors, and HH product consumption should not be regarded as the sole or decisive indicator affecting HAIs.

Limitations of this study:

1. HH product consumption was not analysed concurrently with HH compliance rates, preventing a comprehensive assessment of HCW HH behaviour.
2. The ‘general internal medicine + ICA’ category combined consumption from general wards and intensive care areas, potentially underestimating consumption within the intensive care sub-units of these departments.
3. As a single-centre study, the findings may not be generalizable to different institutions or regions.

Future research should incorporate additional HH compliance metrics and include institutions of varying levels across different regions to more comprehensively evaluate HH practices and their relationship with HAIs.

## CRediT authorship contribution statement

**Hongping Pan:** Writing – review & editing, Writing – original draft, Project administration, Methodology, Formal analysis, Conceptualization. **Chao Liang:** Investigation, Data curation. **Hongjuan Zhu:** Investigation, Data curation. **Hailan Fan:** Visualization, Investigation. **Yu Huang:** Supervision, Project administration, Conceptualization.

## Ethical approval

This study did not require ethics approval.

## Declaration of generative AI and AI-assisted technologies in the writing process

No artificial intelligence (AI) technology was used for any part of this study, including manuscript preparation or revision.

## Funding sources

This research have received funding supports by the Self-funded Project of Yuxi Municipal Science and Technology Plan (Project Number: YF2026022), the Self-funded Project of Yuxi Municipal Science and Technology Plan (Project Number: RKX2026055).

## Conflict of interest statement

The authors declare that they have no conflict of interest.
