# Supplementary material for: Correlation analysis between hand hygiene product consumption trends and healthcare-associated infections in a tertiary comprehensive hospital in Yunnan Province, China (2015–2024)
Source: Infect Prev Pract. 2026 Sep 25;8(4):100594. doi: 10.1016/j.infpip.2026.100594 (PMC13697272; doi:10.1016/j.infpip.2026.100594)
Supplement: Supplementary file 1 [file mmc1.docx]

**APPENDIX A.:**Supplementary materials

Figure S1 Annual growth rate of hand hygiene products consumption by ward (2015-2024)

| Table S1 Daily consumption of HH products and HAI incidence rate in each department from 2015 to 2024 | | | | | | | | | | | | | | | | | |
| --- | --- | --- | --- | --- | --- | --- | --- | --- | --- | --- | --- | --- | --- | --- | --- | --- | --- |
| Year | Total | |  | ICU | |  | NICU | |  | General ward+ICA | |  | General internal medicine | |  | General surgery | |
|  | Consumption per PD(mL/PD） | HAI case **incidence** Rate（%） |  | Consumption per PD(mL/PD） | HAI case **incidence** Rate（%） |  | Consumption per PD(mL/PD） | HAI case **incidence** Rate（%） |  | Consumption per PD(mL/PD） | HAI case **incidence** Rate（%） |  | Consumption per PD(mL/PD） | HAI case **incidence** Rate（%） |  | Consumption per PD(mL/PD） | HAI case **incidence** Rate（%） |
| 2015 | 8.94 | 2.20 |  | 70.47 | 13.12 |  | 85.84 | 0.73 |  | 12.37 | 2.81 |  | 5.89 | 4.22 |  | 7.86 | 1.38 |
| 2016 | 12.71 | 1.84 |  | 98.71 | 18.48 |  | 113.61 | 0.37 |  | 13.76 | 2.20 |  | 11.59 | 3.71 |  | 10.90 | 1.22 |
| 2017 | 12.34 | 1.80 |  | 85.20 | 15.69 |  | 111.88 | 0.59 |  | 12.27 | 2.23 |  | 11.25 | 3.22 |  | 11.22 | 1.25 |
| 2018 | 11.21 | 1.45 |  | 79.09 | 10.12 |  | 133.38 | 0.30 |  | 12.50 | 1.47 |  | 9.09 | 2.77 |  | 9.59 | 1.12 |
| 2019 | 12.50 | 1.53 |  | 92.69 | 13.96 |  | 130.48 | 0.56 |  | 12.95 | 1.56 |  | 11.21 | 2.69 |  | 11.01 | 1.14 |
| 2020 | 15.36 | 1.49 |  | 122.73 | 16.33 |  | 201.52 | 1.05 |  | 14.39 | 2.01 |  | 14.94 | 2.27 |  | 13.27 | 0.91 |
| 2021 | 15.99 | 1.14 |  | 120.55 | 16.19 |  | 278.59 | 0.64 |  | 16.04 | 1.59 |  | 12.27 | 1.71 |  | 14.07 | 0.70 |
| 2022 | 14.73 | 1.07 |  | 117.99 | 15.30 |  | 355.01 | 1.03 |  | 15.10 | 1.67 |  | 12.30 | 1.80 |  | 11.70 | 0.53 |
| 2023 | 13.12 | 1.00 |  | 110.15 | 19.97 |  | 310.23 | 1.21 |  | 12.48 | 1.86 |  | 12.01 | 0.93 |  | 10.51 | 0.61 |
| 2024 | 13.40 | 0.76 |  | 103.30 | 13.85 |  | 326.65 | 1.30 |  | 14.41 | 1.52 |  | 11.24 | 0.80 |  | 10.79 | 0.38 |

Figure S2 HAI case incidence rate trends by ward (2015-2024)
